# Quality of Online Information on Patient-Specific Knee Arthroplasty and Its Impact on Personalized Care

**DOI:** 10.3390/clinpract16010002

**Published:** 2025-12-25

**Authors:** Patrick F. Marko, Lukas K. Kriechbaumer, Marian Mitterer, Sebastian Filipp

**Affiliations:** Department for Orthopedics and Trauma Surgery, Paracelsus Medical University Salzburg, 5020 Salzburg, Austria; l.kriechbaumer@salk.at (L.K.K.); m.mitterer@salk.at (M.M.); s.filipp@salk.at (S.F.)

**Keywords:** patient-specific instrumentation, knee arthroplasty, health literacy, online health information, shared decision-making, patient education

## Abstract

Background: Patient-specific instrumentation (PSI) in total knee arthroplasty represents an increasingly relevant component of personalized surgical planning. As nearly half of orthopedic patients search online for medical information before or after clinical consultation, the quality, accuracy, and readability of publicly available digital resources directly influence patient expectations, shared decision-making, and rehabilitation engagement. This study assessed the content, quality, and readability of online information about PSI in TKA. Methods: Google searches using four predefined PSI-related terms were conducted on 6 March 2025. After applying exclusion criteria, 71 websites were included for evaluation. Websites were categorized as academic or non-academic and analyzed for authorship, reporting of advantages and disadvantages, inaccurate assertions, use of peer-reviewed references, multimedia content, and mention of specific PSI platforms. Website quality was assessed using validated quality evaluation tools (QUEST and JAMA criteria), and readability was evaluated using established readability indices (SMOG, FKGL, and FRE). Results: Academic websites demonstrated significantly higher quality than non-academic sources based on QUEST (25.4 vs. 9.8; *p* < 0.001) and JAMA criteria (3.7 vs. 1.7; *p* < 0.001). Disadvantages of PSI were reported in 69.1% of academic sites versus 12.5% of non-academic sites (*p* < 0.001). Inaccurate claims occurred in 31.3% of non-academic sites but were absent in academic sources (*p* < 0.001). Peer-reviewed references were present in 81.8% of academic websites and only 12.5% of non-academic sites (*p* < 0.001). Readability was uniformly poor across all websites, with no significant group differences (mean SMOG 13.5; mean FKGL 11.8; mean FRE 32.4). Conclusions: Online information about PSI in total knee arthroplasty varies widely in transparency and accuracy, with non-academic websites frequently omitting risks or presenting misleading claims. Given the role of individualized implant planning, accessible and evidence-based digital content is essential to support personalized patient education and shared decision-making. Because limited readability restricts patient comprehension and informed participation in personalized orthopedic care, improving the clarity and accessibility of digital patient resources is essential.

## 1. Introduction

According to previous studies, the demand for total knee arthroplasty (TKA) is expected to increase by 139% by 2040 and 469% by 2060, making total joint arthroplasty (TJA) the most frequently performed orthopedic procedure in the United States [1,2,3,4,5].

Despite the high success rate, the literature shows patient dissatisfaction after TKA to be as high as 20% [6,7,8]. Patient-specific instrumentation (PSI) was developed to improve implant positioning and limb alignment. MRI or CT imaging is used to generate a 3D-printed anatomical model, which serves as the basis for surgical planning and fabrication of patient-specific templates. These cutting blocks guide the oscillating saw intraoperatively, facilitating precise implant placement [9,10,11].

Newly reported results show clear benefits of the PSI technique over conventional instrumentation (CI) regarding the accuracy of implant positioning and a reduced intraoperative blood loss [12,13,14,15,16]. Although PSI aligns conceptually with personalized surgical planning, its clinical benefit remains debated. Evidence for functional superiority over conventional instrumentation is inconsistent, and long-term outcome data are limited. Cost-effectiveness findings also vary across healthcare systems. These uncertainties highlight the importance of accurate and balanced presentation of PSI in publicly accessible online information.

Potential improvements in surgical efficiency do not eliminate the information gap faced by patients. Many seek additional information beyond medical consultations, with approximately 49% of orthopedic patients researching online before and 42% after their appointment [17].

Recent work in orthopedics highlights the growing importance of remote information delivery and its influence on patient involvement in surgical decision-making [18].

The increasing use of telemedicine in orthopedic care further increases patients’ reliance on online health information. Compared with in-person consultations, telemedicine often allows less time for detailed explanation of complex surgical concepts. As a result, poor readability or misleading online content may have a greater negative impact on patient understanding and shared decision-making. The internet has therefore become a central source of medical information. Google remains the most widely used search engine worldwide, with a global market share of approximately 89.8% as of January 2025 [19]. In the health-related context, an estimated 6.5 million searches are performed daily, and approximately 75% of users rely on online sources for medical information [17,20].

The aim of this study was to systematically evaluate the content, quality, and readability of freely accessible online information regarding patient-specific surgical guides for knee arthroplasty. We hypothesized that academic websites would demonstrate higher quality and fewer inaccuracies than non-academic websites and that overall readability would fall below recommended levels for patient education.

## 2. Materials and Methods

### 2.1. Search Strategy

A structured Google search was conducted on 6 March 2025 using the terms “PSI knee,” “PSI total knee arthroplasty”, “patient-specific instrumentation knee”, and “patient-specific instrumentation total knee arthroplasty”. These terms were selected because they reflect the most used descriptors in the clinical literature and in routine orthopedic communication. Broader variants such as “custom cutting guides” or “patient-matched guides” were deliberately not included, as preliminary testing showed that these terms frequently retrieved content unrelated to patient-specific instrumentation for knee arthroplasty, reducing specificity.

All searches were performed through www.google.com/ncr (accessed on 6 March 2025) to prevent automatic geographic redirection. The browser was set to private mode, and no virtual private network (VPN) was used. Searches were executed from a server located in Salzburg, Austria, which may influence ranking results; the location is reported here to ensure reproducibility.

For each of the four search terms, the first 50 results were collected. The decision to restrict the dataset to the first 50 results per query was based on evidence indicating that over 90% of users do not view results beyond the first five pages of Google search output [21]. Including deeper-ranked websites would therefore be less representative of actual patient information-seeking behavior and would risk over-sampling low-visibility content.

### 2.2. Eligibility Criteria

After removing duplicates, websites were screened for eligibility. Exclusion criteria included: Absence of written content, content behind paywalls or login requirements, websites unrelated to patient-specific instrumentation, pages discussing only conventional knee arthroplasty, and links directing exclusively to video platforms (e.g., YouTube, VuMedi).

Video-based platforms were excluded because the objective of this study was to evaluate written patient-facing information. Although multimedia content plays an increasing role in patient education, written text remains the primary format encountered on institutional and medical-practice websites and is the basis for established readability scoring systems. The implications of this exclusion are addressed in the discussion.

**Figure 1:**
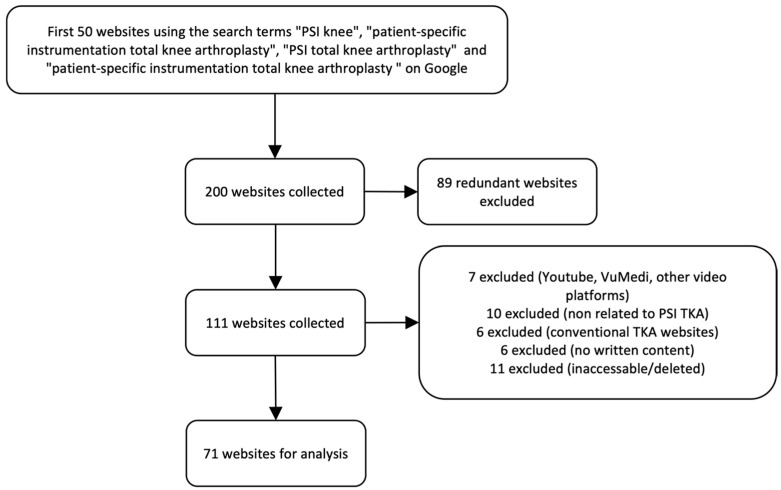
Flow chart of the exclusion process for website selection. TKA, total knee arthroplasty.

Following the application of all criteria, 71 websites remained for evaluation. The selection process is summarized in Figure 1.

### 2.3. Website Screening Procedure

Only primary landing pages were analyzed to standardize data collection. Pop-up confirmations or CAPTCHA steps were completed as required, but no submenu or linked subpage content was systematically evaluated.

Three reviewers with different levels of clinical experience independently screened all websites: a senior orthopedic surgeon, an orthopedic specialist, and a fifth-year orthopedic resident. Prior to data extraction, all reviewers received standardized instructions on applying the scoring instruments and operational definitions. Reviewer agreement was high, with discrepancies occurring in only five websites, mainly concerning fulfillment of Journal of American Medical Association (JAMA) benchmark criteria. These cases were resolved through a structured consensus discussion led by the senior author. Formal inter-rater reliability metrics were not calculated because the study aimed to descriptively characterize online content rather than validate an assessment instrument; however, the absence of reliability statistics is acknowledged as a methodological limitation.

The content of each website was examined for mention of the following criteria: authorship, potential advantages or disadvantages of PSI in TKA, inaccurate assertions, peer-reviewed references, video/animations, or specific PSI platforms.

We used the QUality Evaluation Scoring Tool (QUEST Score) [22] as a quantitative testing instrument, and the JAMA benchmark criteria as a more simplistic model for evaluating quality of online material [23,24].

Readability was examined using 3 established scoring tools: the Simple Measure of Gobbledygook (SMOG) Readability Index [25], the Flesch-Kincaid Grade Level (FKGL) [26], and the Flesch Reading Ease Formula (FRE) [27]. All objective scores were then averaged between the 3 reviewers.

### 2.4. Authorship

All included websites were examined for authorship and content ownership and categorized using predefined operational criteria. Academic websites were defined as those hosted by universities, teaching hospitals, professional societies, or peer-reviewed journals. Commercial websites included manufacturer-operated, industry-sponsored, or marketing-driven platforms. Non-academic medical practice websites were defined as clinic- or physician-operated pages without formal academic affiliation. Government websites were identified as those maintained by national or regional public health institutions.

### 2.5. Potential Benefits of PSI in TKA

The mention of potential benefits of PSI over CI in TKA, such as improved implant placement, reduced intraoperative blood loss, and an overall reduced surgery duration [12,13,14,15,16], was reviewed separately for each website.

### 2.6. Potential Disadvantages of PSI in TKA

We also checked every website for the potential disadvantages of PSI, e.g., increased costs to be assumed by the patient, necessity of an additional CT or MRI scan, or the lack of verification tools intraoperative [28].

### 2.7. Inaccurate Assertions

Statements and phrases implying a strong level of certainty, like “will” instead of “may” or “can” were considered inaccurate assertions (e.g., patient-specific instrumentation will result in a shorter hospitalization compared to conventional instrumentation).

In addition, claims about PSI that could not be clearly confirmed by current studies were investigated. These included a faster return to normal activity, a shorter hospitalization, smaller incisions, less pain levels, or a longer implant durability.

### 2.8. Existence of Peer-Reviewed References

The use of at least one peer-reviewed reference was documented as a further quality attribute.

### 2.9. Content

All websites were examined for the presence of informative videos and/or animations. In addition, it was documented whether specific PSI platforms from different manufacturers were mentioned, such as Persona/NexGen PSI (Zimmer Biomet, Warsaw, IN, USA), Trumatch (DePuy Synthes, Raynham, MA, USA), MyKnee (Medacta, Castel San Pietro, Switzerland), Visionaire (Smith + Nephew, London, UK), or other solutions.

### 2.10. Website Quality

As overall indicators for quality of the websites, we used the QUality Evaluation Scoring Tool (QUEST) score and the Journal of American Medical Association (JAMA) benchmark criteria.

QUEST is a validated, seven-item tool that rates authorship, attribution, conflict of interest, currency, references, tone, and bias. Scores range from 0 to 28, with higher values indicating better quality of health information and alignment with patient-physician communication principles [22].

Another valid and user-friendly tool for the assessment of quality are the JAMA benchmark criteria, which uses four core standards to evaluate websites: authorship, attribution, disclosure, and currency. The exact definitions of the JAMA benchmark criteria are mentioned by Silberg et al. in their publication from 1997 [23]. Being the most streamlined of the quality assessment tools, the JAMA benchmark allows the evaluator to quickly detect websites that lack the most basic components of information transparency and reliability. Therefore, it cannot be expected to perform as comprehensive an assessment as other, more sophisticated models [24].

### 2.11. Readability

General readability was assessed using three validated readability indices. Using multiple measures enhances robustness by minimizing metric-specific bias.

The Simple Measure of Gobbledygook (SMOG) Readability Index measures text comprehensibility based on the number of polysyllabic words. It estimates the education level required to understand the text and is commonly used in health communication and academic writing [25].

The Flesch-Kincaid Grade Level estimates the U.S. school grade needed to understand a text. It is based on average sentence length and syllables per word. Higher scores indicate greater complexity and educational demand [26].

The Flesch Reading Ease (FRE) score assesses readability based on sentence length and syllables per word. Scores range from 0–100, with higher values indicating easier texts: 90–100 (very easy), 60–70 (standard), 30–50 (difficult), and 0–30 (very difficult, academic) [27].

Readability was assessed using the online tool Readable.com (Readable Ltd., Hassocks, UK; accessed April 2025). When direct URL analysis was not feasible, a coherent text excerpt of at least ten sentences was analyzed to obtain readability scores—preferably forming a coherent passage.

### 2.12. Data Analyses

Data were collected into Excel and analyzed using SPSS Statistics (version 31, IBM, Armonk, NY, USA). Normality of continuous variables was assessed using the Shapiro–Wilk test. Given the distributions observed, group differences in continuous variables were analyzed with independent samples *t*-tests. Categorical variables were compared using Fisher’s exact test.

Statistical significance was defined as *p* < 0.05 (two-sided). To improve interpretability, proportions are reported as percentages and selected group comparisons include 95% confidence intervals.

## 3. Results

A total of 71 websites were left for review after duplicate web sites were excluded. These comprised 55 academic sites (77.5%), 4 commercial sites (5.6%), and 12 non-academic medical practice sites (16.9%). Subgroup analysis was not performed due to the small and unbalanced sample sizes of commercial websites and medical-practice websites, which would limit statistical reliability. Therefore, both subgroups were analyzed together as a non-academic group, with differences described qualitatively.

To test the association between group membership (academic vs. non-academic) and the proportion of mentioned benefits, Fisher’s Exact Test was conducted. The results show that there is no significant relationship between group and benefits, *p* = 0.402 (two-tailed). The proportion in the academic group is higher at 98.2%, compared to 93.8% in the non-academic group. However, the difference is not statistically significant.

### 3.1. Website Content Characteristics

The potential disadvantages of PSI were mentioned on academic websites in 69.1% of cases, compared to only 12.5% on non-academic websites (*p* < 0.001).

Inaccurate claims were never made on academic websites, while this was the case for 31.3% of non-academic websites (*p* < 0.001).

81.8% of academic websites used at least one peer-reviewed reference, whereas this was only true for 12.5% of non-academic websites (*p* < 0.001).

Videos were used on 50.0% of non-academic websites and 36.4% of academic websites (*p* = 0.243).

References to PSI platforms from other providers were made by 37.5% of non-academic websites and 21.8% of academic websites (*p* = 0.172). The results regarding various website contents are summarized in Table 1.

### 3.2. Quality Assessment Results

**Figure 2:**
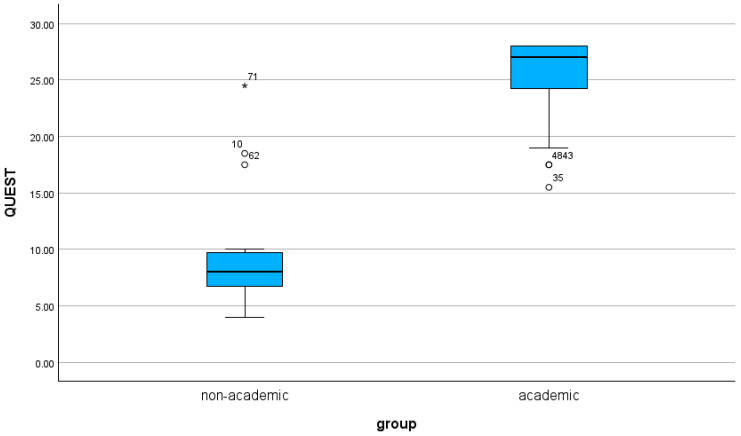
The results for the QUEST Score based on authorship are shown as a boxplot.

**Figure 3:**
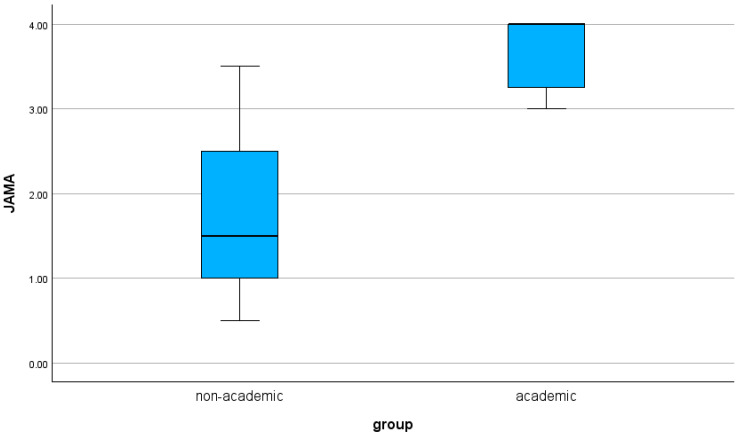
The results for the JAMA Score based on authorship are shown as a boxplot.

Academic websites demonstrated significantly higher quality scores than non-academic websites across both assessment tools. The mean QUEST score was 25.4 (range: 15.5–28.0) for academic sources and 9.8 (range: 4.0–24.5) for non-academic sources (*p* < 0.001). Similarly, academic websites achieved higher JAMA benchmark scores (mean 3.7, range: 3.0–4.0) compared with non-academic websites (mean 1.7, range: 0.5–3.5; *p* < 0.001). The distribution of scores is illustrated in Figure 2 and Figure 3.

### 3.3. Readability Results

The readability of the websites was assessed using three indices: SMOG, FKGL, and FRE. The readability differed only slightly for all three scores measured (SMOG: mean 13.9 vs. 13.1, *p* = 0.317; FKGL: mean 12.1 vs. 11.5, *p* = 0.358; FRE: mean 32.4 vs. 32.3, *p* = 0.981).

The results for quality and readability are summarized in Table 2 and Table 3, as well as in Figure 2 and Figure 3.

### 3.4. Subgroup Description: Commercial vs. Medical-Practice Websites

Although commercial and medical-practice websites were analyzed together as a non-academic group, descriptive differences were observed. Commercial websites more often mentioned specific patient-specific instrumentation platforms and used promotional language, whereas medical-practice websites more frequently provided procedural information but rarely cited peer-reviewed sources. Both subgroups similarly omitted disadvantages and presented inaccurate or overly certain claims.

## 4. Discussion

This study analyzed the quality, content, and readability of freely accessible online information about patient-specific instrumentation used in total knee arthroplasty. The findings demonstrate marked differences between academic and non-academic websites, with substantial implications for personalized orthopedic care and patient education.

### 4.1. Quality and Transparency of Information

Academic websites consistently achieved higher QUEST and JAMA quality scores, reflecting clearer attribution, greater transparency, and better alignment with evidence-based communication standards. In contrast, non-academic websites frequently omitted essential information such as disadvantages or limitations of patient-specific instrumentation and, in several cases, presented inaccurate or overly deterministic claims. These trends are consistent with findings from prior studies showing that commercial or self-promotional online health content often emphasizes benefits while minimizing risks [29,30].

A key concern is the underreporting of PSI disadvantages on non-academic websites—only 12.5% addressed them versus 69.1% of academic sites. Such omissions undermine balanced risk-benefit communication, a core element of informed consent, and may contribute to unrealistic expectations and poor decision-making among patients [31]. This systematic asymmetry is consistent with patterns observed in other commercially influenced online health materials, where selective presentation of benefits is common [32,33].

A notable proportion of non-academic sites also referenced specific instrumentation platforms, a practice most prevalent among commercially affiliated sources. This may reflect marketing-driven motivations to highlight a particular technology, further contributing to incomplete or biased informational content. The lack of disclosure statements or supporting references on these sites amplifies the risk of misinformation. Strengthening transparency standards for online orthopedic information, including explicit declarations of commercial interests, may help mitigate this issue.

### 4.2. Digital Health Literacy and Its Role in Personalized Orthopedic Care

Online health information now plays a central role in shaping patient perceptions, particularly as personalized and technology-enhanced surgical approaches become more common. Many patients search for information before or after clinical consultation, and telemedicine-based interactions further increase reliance on digital sources. In this evolving context, high-quality online information can support shared decision-making by enabling patients to better understand individualized surgical strategies, anticipate realistic outcomes, and articulate expectations more effectively.

However, information that is biased, incomplete, or clinically inaccurate may undermine this process. When critical disadvantages of patient-specific instrumentation are omitted, such as the need for additional imaging or the lack of intraoperative verification tools, patients may develop unrealistic expectations or an incomplete understanding of trade-offs. Similarly, inaccurate assertions regarding recovery, pain reduction, or implant longevity can distort evidence-based discussions and may contribute to postoperative dissatisfaction.

### 4.3. Readability Barriers: A Major Obstacle for Effective Personalized Care

Readability scores across all websites were uniformly poor, with average SMOG and Flesch-Kincaid Grade Level values corresponding to upper secondary or early college reading levels. These scores substantially exceed recommended health communication standards, which advise sixth-to eighth grade reading levels for general patient materials [34].

For PSI, elevated SMOG and FKGL scores may impair patient understanding of procedure-specific concepts, such as the need for preoperative CT or MRI imaging, the rationale behind individualized alignment strategies, or the distinction between patient-specific cutting guides and conventional instrumentation. Limited comprehension of these aspects may reduce patient preparedness for consultation, compromise informed consent, and hinder meaningful participation in personalized surgical planning.

To address these challenges, several practical solutions should be considered:

- Plain-language summaries that distill essential information into concise, patient-friendly terms.
- Visual aids, including diagrams, animations, and comparison graphics, to simplify complex biomechanical and procedural concepts.
- Co-designed educational materials, developed with patient input to ensure clarity, accessibility, and alignment with real-world informational needs.
- Standardized, evidence-based institutional resources, ideally distributed through orthopedic societies or academic departments, to counterbalance commercially biased online content.

These strategies may improve digital health literacy and support more effective shared decision-making in personalized orthopedic care.

### 4.4. Clinical and Educational Implications

The findings underscore several practical implications for clinicians and institutions. Healthcare providers should proactively guide patients toward high-quality, academically vetted resources and address misconceptions originating from commercial content. Orthopedic departments and professional societies may consider creating standardized, evidence-based online materials to support accurate patient understanding of modern surgical technologies.

Beyond textual simplification, visual enhancements such as diagrams, animations, and explanatory videos may improve comprehension of biomechanical principles underpinning patient-specific instrumentation. Incorporating patient feedback or co-designing educational content can also ensure that learning materials reflect real-world patient needs and reduce ambiguity. These strategies align with contemporary models of telemedicine and digital health literacy.

### 4.5. Study Strengths

This study has several strengths that enhance the relevance and reliability of its findings. It is one of the first analyses to focus specifically on online information about patient-specific knee instrumentation, a fast-evolving surgical technology. The use of validated quality and readability tools ensures rigorous assessment across multiple dimensions, and the inclusion of reviewers with different clinical experience levels enhances the robustness of website evaluation. Moreover, by focusing on the highest-ranking Google results, the analysis reflects real-world information-seeking behavior and provides insight into the content most likely encountered by patients.

### 4.6. Limitations and Future Research

Several limitations must be acknowledged. This approach may limit external validity, as patients increasingly access medical information through video-based and social media platforms not included in this analysis. Although the Google-based strategy reflects common text-based information-seeking behavior, the growing role of multimedia resources suggests that the findings may not capture the full spectrum of online patient information. Inter-rater reliability metrics were not calculated, although consensus procedures were used to resolve scoring disagreements. Additionally, readability formulas assess linguistic complexity but do not account for layout, visual design, or multimedia integration, all of which may influence comprehension.

Future research should explore the quality and comprehensibility of video-based educational platforms, assess patient comprehension directly through user-centered testing, and examine temporal changes in online content as personalized instrumentation technologies become more widely implemented. Comparative analyses across languages or countries may also reveal differences in digital orthopedic education. Furthermore, evaluating co-designed instructional materials could provide insights into how patient collaboration improves clarity and accessibility.

## 5. Conclusions

This study reveals substantial variability in the quality, transparency, and readability of online information about patient-specific instrumentation in total knee arthroplasty. Academic websites consistently provided more accurate, evidence-based, and balanced content, whereas non-academic sources frequently omitted key disadvantages or presented misleading claims. Across all website types, readability was poor, limiting accessibility for many patients.

As PSI becomes increasingly integrated into personalized surgical planning, patients require reliable and comprehensible online resources to support informed decision-making and individualized rehabilitation strategies. Enhancing the clarity, accuracy, and accessibility of digital patient education is therefore essential to advancing patient-centered orthopedic care. Future work should focus on developing standardized, high-quality online materials and integrating digital health literacy principles into orthopedic patient communication.

## Figures and Tables

**Table 1:** Content of Websites Based on Authorship.

	Group		
Content	Non-Academic	Academic	*p*-Value
benefits	93.8%	98.2%	0.402
disadvantages	12.5%	69.1%	<0.001
inaccurate assertions	31.3%	0.0%	<0.001
peer-reviewed references	12.5%	81.8%	<0.001
videos	50.0%	36.4%	0.243
PSI platforms	37.5%	21.8%	0.172

**Table 2:** Quality Scores Based on Authorship.

	Group				
	Non-Academic		Academic		
Score	Mean	Range	Mean	Range	*p*-Value
QUEST	9.8	4.0–24.5	25.4	15.5–28.0	<0.001
JAMA	1.7	0.5–3.5	3.7	3.0–4.0	<0.001

**Table 3:** Readability Scores Based on Authorship.

	Group				
	Non-Academic		Academic		
Score	Mean	Range	Mean	Range	*p*-Value
SMOG	13.9	9.8–19.0	13.1	9.3–18.1	0.317
FKGL	12.1	8.8–16.8	11.5	6.0–16.3	0.358
FRE	32.4	11.1–54.4	32.3	10.7–60.2	0.981

## Data Availability

Any further data will be available on a reasonable request from the corresponding author via email (p.marko@salk.at).

## Abbreviations

The following abbreviations are used in this manuscript:

CI	Conventional Instrumentation
CT	Computed Tomography
FKGL	Flesch-Kincaid Grade Level
FRE	Flesch Reading Ease
JAMA	Journal of the American Medical Association
MRI	Magnetic Resonance Imaging
PSI	Patient-Specific Instrumentation
QUEST	QUality Evaluation Scoring Tool
SMOG	Simple Measure of Gobbledygook
SPSS	Statistical Package for the Social Sciences
TJA	Total Joint Arthroplasty
TKA	Total Knee Arthroplasty
